# Single-cell profiling reveals distinct subsets of CD14+ monocytes drive blood immune signatures of active tuberculosis

**DOI:** 10.3389/fimmu.2022.1087010

**Published:** 2023-01-11

**Authors:** Hannah Hillman, Nabeela Khan, Akul Singhania, Paige Dubelko, Ferran Soldevila, Rashmi Tippalagama, Aruna D. DeSilva, Bandu Gunasena, Judy Perera, Thomas J. Scriba, Cynthia Ontong, Michelle Fisher, Angelique Luabeya, Randy Taplitz, Gregory Seumois, Pandurangan Vijayanand, Catherine C. Hedrick, Bjoern Peters, Julie G. Burel

**Affiliations:** ^1^ Center for Infectious Disease and Vaccine Research, La Jolla Institute for Immunology, La Jolla, CA, United States; ^2^ Department of Paraclinical Sciences, Faculty of Medicine, General Sir John Kotelawala Defence University, Colombo, Sri Lanka; ^3^ Medical Unit, National Hospital for Respiratory Diseases, Welisara, Sri Lanka; ^4^ South African Tuberculosis Vaccine Initiative, Institute of Infectious Disease and Molecular Medicine and Division of Immunology, Department of Pathology, University of Cape Town, Cape Town, South Africa; ^5^ Department of Medicine, City of Hope National Medical Center, Duarte, CA, United States; ^6^ Department of Medicine, University of California San Diego, La Jolla, CA, United States; ^7^ Center for Autoimmunity and Inflammation, La Jolla Institute for Immunology, La Jolla, CA, United States

**Keywords:** tuberculosis, monocytes, transcriptomics (RNA-Seq), flow cytometry, immune signatures

## Abstract

**Introduction:**

Previous studies suggest that monocytes are an important contributor to tuberculosis (TB)-specific immune signatures in blood.

**Methods:**

Here, we carried out comprehensive single-cell profiling of monocytes in paired blood samples of active TB (ATB) patients at diagnosis and mid-treatment, and healthy controls.

**Results:**

At diagnosis, ATB patients displayed increased monocyte-to-lymphocyte ratio, increased frequency of CD14+CD16- and intermediate CD14+CD16+ monocytes, and upregulation of interferon signaling genes that significantly overlapped with previously reported blood TB signatures in both CD14+ subsets. In this cohort, we identified additional transcriptomic and functional changes in intermediate CD14+CD16+ monocytes, such as the upregulation of inflammatory and MHC-II genes, and increased capacity to activate T cells, reflecting overall increased activation in this population. Single-cell transcriptomics revealed that distinct subsets of intermediate CD14+CD16+ monocytes were responsible for each gene signature, indicating significant functional heterogeneity within this population. Finally, we observed that changes in CD14+ monocytes were transient, as they were no longer observed in the same ATB patients mid-treatment, suggesting they are associated with disease resolution.

**Discussion:**

Together, our study demonstrates for the first time that both intermediate and classical monocytes individually contribute to blood immune signatures of ATB and identifies novel subsets and associated gene signatures that may hold disease relevance.

## Introduction

Tuberculosis (TB) is a leading cause of mortality from infectious diseases worldwide. The WHO estimates that one-quarter of the world population is infected with *Mtb*, with 10 million new cases and 1.5 million deaths each year (1). In 2020, the annual number of TB deaths has risen for the first time in more than a decade, as many cases have gone undiagnosed or untreated during the numerous COVID-19 related lockdowns worldwide (1).

Upon infection with *Mtb*, most individuals are asymptomatic and control bacilli within lung granulomas, or even eliminate the infection altogether, whereas others will exhibit *Mtb* multiplication primarily in the lung, causing active pulmonary disease (2, 3). The asymptomatic stage of *Mtb* infection is typically diagnosed with positive reactivity to Interferon Gamma Release Assays (IGRA) tests in the blood (2). Active TB (ATB) is associated with clinical symptoms, risk of transmission, and high mortality (2). Research on host immune responses to *Mtb* has been ongoing for decades, yet we still do not have a full understanding of which cellular and molecular components constitute a protective (associated with controlled infection, or even sterilization) versus a pathologic (leading to the development of active disease) immune response (4–6).

The transcriptomic analysis of immune cell populations is a powerful approach for the identification of mechanistic signatures of disease, including TB (7, 8). Numerous studies have identified whole blood and peripheral blood mononuclear cells (PBMC) signatures of TB, as reviewed in (9–11). Such signatures have proven to be promising tools for improving diagnosis of ATB, particularly in the presence of co-morbidities such as HIV (12, 13), predicting which IGRA+ individuals will progress to active disease (11, 14, 15), and predicting TB treatment outcomes (16, 17). Different blood cell types are likely responsible for different component of these signatures. The seminal blood signature of ATB associated with IFN signaling has been shown to be expressed by neutrophils and to a lower extent by CD14+ monocytes (9, 18), which are present in abundance in the blood. Conversely, blood signatures of early progression from IGRA+ to ATB were enriched for NK cell and T cell modules (15), and blood signatures of anti-TB treatment shared between ATB and IGRA+ individuals at risk of progression were associated with activated T cells (19).

There is evidence that monocytes can be directly infected by *Mtb in vitro* (20), and participate in the host immune response to infection (21, 22). Monocytes are also precursors to interstitial macrophages that are recruited to the lung during *Mtb* infection (22, 23). Moreover, total monocyte counts as well as monocyte to lymphocyte ratio are increased in ATB compared to IGRA+ (24, 25), are prospective markers of ATB risk (26, 27), and are reduced following TB therapy in ATB (25). Altogether, there is large amount of evidence suggesting that monocytes are an important contributing subset to blood immune signatures in TB.

Functionally, monocytes are known to possess phagocytic and pathogen sensing abilities. In humans, three populations of monocytes with unique functional properties can be identified by surface expression of CD14 and CD16 (28). Classical monocytes (CD14^+^CD16^-^) produce a multitude of pro-inflammatory cytokines and are known for their superior phagocytic ability, while non-classical monocytes (CD14^-^CD16^+^) are regarded for their role in transendothelial crawling and adhesion, which aid in anti-viral response (29, 30). The intermediate population, commonly defined as CD14^+^CD16^+^, holds the highest antigen presentation capability amongst monocytes, and can produce reactive oxygen species and IL-12 (30).

In this study, we carried out a comprehensive cellular and gene profiling of the circulating monocyte compartment in the context of *Mtb* infection. Using flow cytometry, bulk and single-cell RNA sequencing, we isolated and interrogated the transcriptomic profile of monocyte subsets isolated from PBMC in a cohort of ATB patients with paired sampling at diagnosis and following treatment (i.e., two to six months after initiation of a standard six-month anti-TB therapy), as well as from *Mtb* sensitized (IGRA+) and unsensitized (IGRA-) healthy individuals. We aimed to compare the frequency and gene signature of each subset in ATB at diagnosis (uncontrolled infection) compared to treated ATB, IGRA+ (successful control of infection), and IGRA- (no infection) to further inform on the contribution of myeloid cells to blood TB signatures and provide novel molecular insights into myeloid-associated immune responses that may be associated with the control of *Mtb*.

## Results

### The increased frequency of circulating monocytes in ATB patients stems from classical CD14^+^CD16^-^ and intermediate CD14^+^CD16^+^ monocytes

**Figure 1:**
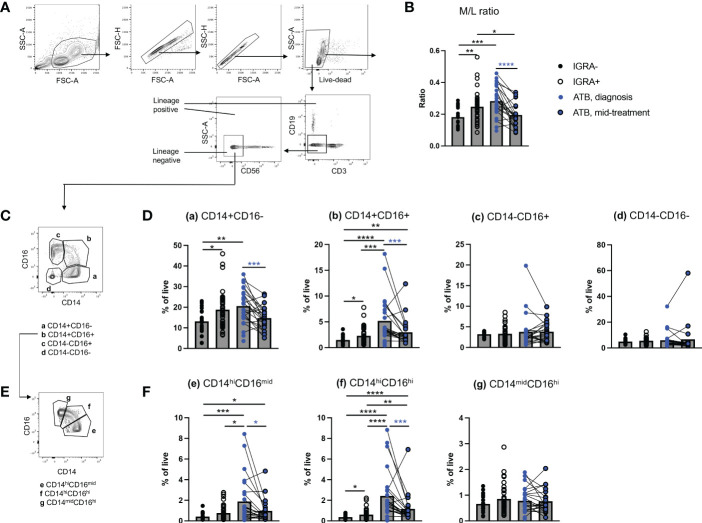
Increased frequency of circulating classical CD14^+^CD16^-^ and intermediate CD14^+^CD16^+^ monocytes in ATB at diagnosis. **(A)** Gating strategy to calculate the monocyte to lymphocyte (M/L) ratio based on the negative or positive expression of lymphocyte lineage markers using flow cytometry. **(B)** M/L ratio in active TB (ATB) at diagnosis, ATB mid-treatment, IGRA+ and IGRA- cohorts. **(C)** Gating strategy to identify CD14/CD16 sub-populations from the lymphocyte lineage negative population identified in Figure 1A. **(D)** Frequency of lineage negative CD14^+^CD16^-^, CD14^+^CD16^+^, CD14^-^CD16^+^ and CD14^-^CD16^-^ cells in ATB diagnosis, ATB mid-treatment, IGRA+ and IGRA- cohorts. **(E)** Gating strategy to identify CD14/CD16 sub-populations within intermediate CD14^+^CD16^+^ monocytes identified in Figure 1C. **(F)** Frequency of CD14^hi^CD16^mid^, CD14^hi^CD16^hi^ and CD14^mid^CD16^hi^ monocytes in ATB diagnosis, ATB mid-treatment, IGRA+ and IGRA- cohorts. Data was from cryopreserved PBMC of 25 ATB subjects at diagnosis (with 22 paired mid-treatment samples), 40 IGRA+ and 20 IGRA- individuals. *p < 0.05, **p < 0.01, ***p < 0.001, ****p < 0.0001, nonparametric unpaired Mann-Whitney U test (black stars) and nonparametric paired Wilcoxon test (blue stars).

For this study, we utilized cryopreserved PBMC of 20 healthy IGRA-, 40 IGRA+, and 25 ATB patients (collected at diagnosis, including 22 with a mid-treatment paired sample) as described in the methods section and in **Table S1**. Mean age was 33, 39 and 34 years old for the IGRA-, IGRA+ and ATB cohorts, respectively (**Table S1**). The proportion of female to male was 60%, 45% and 40% for the IGRA-, IGRA+ and ATB cohorts, respectively (**Table S1**). To confirm previous findings that the M/L ratio was elevated in ATB at diagnosis (24, 25), we first quantified the proportion of monocytes and lymphocytes using flow cytometry. Lymphocytes were identified as positive- and monocytes were identified as negative for the expression of any of the three major lymphocyte markers CD3, CD19 and CD56 (lineage positive and lineage negative populations, **Figure 1A**). The M/L ratio was calculated by dividing the frequency of live lineage negative cells with the frequency of live lineage positive cells. The M/L ratio was increased in PBMC samples from ATB patients at diagnosis and to a lesser extent in IGRA+ individuals, in comparison to the IGRA- healthy cohort (**Figure 1B**). The high M/L ratio in ATB patients at diagnosis was reduced in longitudinal samples collected mid-treatment, reverting to levels similar to those in the IGRA- healthy cohort (**Figure 1B**). The significance of this finding was confirmed using a complementary gating strategy identifying monocytes and lymphocytes using size and complexity parameters (FSC and SSC), without taking in consideration the expression of lineage markers (M/L size ratio, **Figures S1A and S1B**).

To investigate which monocyte subset contributed to the elevated M/L ratio in ATB at diagnosis, we further identified amongst lineage negative (CD3^-^CD19^-^CD56^-^) cells the four major myeloid populations present in peripheral blood, namely: classical CD14^+^CD16^-^, intermediate CD14^+^CD16^+^, non-classical CD14^-^CD16^+^ monocytes, and CD14^-^CD16^-^ cells (a, b, c, and d in **Figure 1C**). The CD14^-^CD16^-^ population is expected to contain non-monocyte myeloid cells present in PBMC, such as dendritic cells and granulocytes (e.g., basophils) (31, 32). The frequency of classical CD14^+^CD16^-^ and intermediate CD14^+^CD16^+^ monocytes was increased in both ATB diagnosis and IGRA+ cohorts, compared to the IGRA- cohort (**Figure 1D**). The frequency of intermediate CD14^+^CD16^+^ monocytes was further increased in ATB at diagnosis compared to IGRA+ (**Figure 1D**). In ATB patients, the frequencies of classical CD14^+^CD16^-^ and intermediate CD14^+^CD16^+^ monocytes were reduced at mid-treatment compared to diagnosis (**Figure 1D**). No significant changes in the frequency of non-classical CD14^-^CD16^+^ monocytes or CD14^-^CD16^-^ myeloid cells were observed between cohorts or during anti-TB therapy (**Figure 1D**).

Previous transcriptomic studies have shown that the intermediate CD14^+^CD16^+^ monocyte population is heterogeneous and overlaps with classical CD14^+^CD16^-^ and non-classical CD14^-^CD16^+^ monocytes (33). Since CD14 and CD16 display a continuous ‘smeary’ expression in myeloid cells, the CD14^+^CD16^+^ population may contain ‘contaminating’ CD14^+^CD16^-^ or CD14^-^CD16^+^ monocytes depending on where CD14 and CD16 positive expression gates are set (34). To examine this possible ‘contamination’, we divided the CD14^+^CD16^+^ population into three sub-populations: CD14^hi^CD16^mid^, CD14^hi^CD16^hi^ and CD14^mid^CD16^hi^ (e, f, and g in **Figure 1E**). Both CD14^mid^CD16^hi^ and CD14^hi^CD16^hi^ cell subsets showed increased frequency in ATB patients at diagnosis compared to mid-treatment and IGRA-/+ controls (**Figure 1F**). The significance was stronger for CD14^hi^CD16^hi^ cells, suggesting that the increased frequency observed in intermediate CD14^+^CD16^+^ monocytes in ATB at diagnosis is not due to a poor separation between CD14/CD16 positive and negative populations, but rather reflects a ‘true’ increased frequency of cells co-expressing these two markers.

Taken together, our data show that the increased frequency of circulating monocytes found in ATB patients at diagnosis stems from classical CD14^+^CD16^-^ and intermediate CD14^+^CD16^+^ monocytes. These changes were reverted following 2-3 months of anti-TB therapy.

### Identification of transcriptomic modules that can distinguish classical CD14^+^CD16^-^ from intermediate CD14^+^CD16^+^ monocytes and display dysregulated expression in ATB at diagnosis

**Figure 2:**
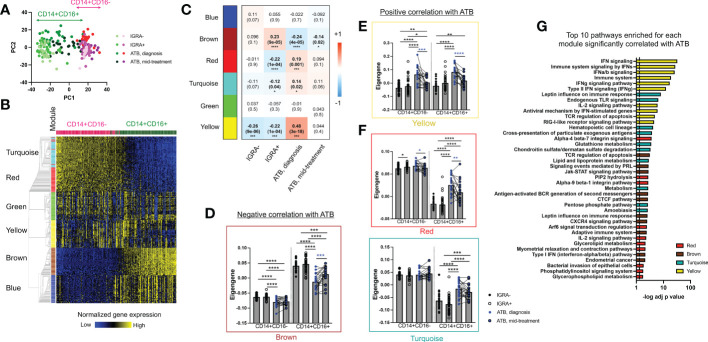
The CD14^+^CD16^+^ population in ATB at diagnosis shows the highest transcriptomic changes compared to other cell populations and cohorts. **(A)** Principal component analysis of the transcriptome of CD14^+^CD16^+^ and CD14^+^CD16^-^ myeloid cells in active TB (ATB) at diagnosis, ATB mid-treatment, IGRA+ and IGRA- cohorts using their combined 1,000 most variable genes. Cells were sorted as defined in **Figure 1C** and their transcriptomic profile defined by RNA sequencing. **(B)** Modular analysis of CD14^+^CD16^+^ and CD14^+^CD6^-^ cells using their combined 3,000 most variable genes and the WGCNA package (35). **(C)** Module trait-relationship analysis showing the Pearson correlation coefficient (and associated p-value in brackets) between each module and clinical cohort groups. Significant correlations are represented in bold. **(D-F)** Eigengene values for each module in ATB diagnosis, ATB mid-treatment, IGRA+ and IGRA- cohorts in CD14^+^CD16^+^ and CD14^+^CD6^-^ cells. Statistical comparisons between eigengene values were performed with nonparametric unpaired Mann-Whitney U test (black stars) and nonparametric paired Wilcoxon test (blue stars). **(G)** Pathway enrichment analysis for the yellow, turquoise, red and brown modules identified in **Figure 2** which showed significant changes in expression in ATB at diagnosis compared to IGRA+/- cohorts. The top 10 pathways are displayed, ranked by their decreasing p-value and the full list of pathways for each module is available in **Table S5**. Data was from cryopreserved PBMC of 25 ATB subjects at diagnosis (with 22 paired mid-treatment samples), 40 IGRA+ and 20 IGRA- individuals. *p < 0.05, **p < 0.01, ***p < 0.001, ****p < 0.0001.

Next, we aimed to identify whether the increased frequency in classical CD14^+^CD16^-^ and intermediate CD14^+^CD16^+^ monocytes in ATB at diagnosis was only quantitative, or if we could also identify qualitative transcriptomic changes in these two populations. Using the same gating strategy as for flow cytometry, classical CD14^+^CD16^-^ and intermediate CD14^+^CD16^+^ monocytes were sorted and processed for bulk RNA sequencing. As expected, principal component analysis (PCA) of the most variable genes segregated samples by cell type (**Figure 2A**). The intermediate CD14^+^CD16^+^ monocyte population showed higher diversity, as indicated by its wider spread over the PC1 component, compared to classical CD14^+^CD16^-^ monocytes (**Figure 2A**).

Differential expression analysis identified thousands of genes significantly dysregulated in ATB at diagnosis compared to other cohorts, in both monocyte subsets. To reduce the analysis dimensionality and identify gene clusters with consistent co-expression patterns, we performed a modular analysis on the dataset using the well-established WGCNA algorithm (35). Modular analysis groups together genes with a similar expression pattern within a given sample, into modules. Since it reduces the dimensionality of the dataset from thousands of genes to a handful of modules, it is also very useful to perform correlations with categorical traits, such as a disease condition. Modular analysis on the sorted CD14+ monocytes bulk RNA sequencing dataset identified six modules (**Figure 2B**, individual gene list for each module is available in **Table S3**). The turquoise (1,348 genes) and red (39 genes) modules showed highest expression in classical CD14^+^CD16^-^ monocytes, whereas the brown (427 genes) and blue (914 genes) modules showed the highest expression in intermediate CD14^+^CD16^+^ monocytes (**Figure 2B**). The green (46 genes) and yellow (65 genes) modules showed similar expression levels across both subsets (**Figure 2B**). The remaining 161 genes were not classified into any module.

Next, we explored the association between each module and our disease cohorts using a module-trait relationship analysis (**Figure 2C**). We identified one module (brown) with positive correlation with IGRA+ and negative correlation with ATB at diagnosis and ATB mid-treatment (Pearson correlation coefficient = 0.23, -0.24 and -0.14, and *p* value = 9e-05, 4e-05 and 0.02, for IGRA+, ATB diagnosis and ATB mid-treatment, respectively). Three modules (red, turquoise and yellow) showed a positive correlation with ATB at diagnosis (Pearson correlation coefficient = 0.19, 0.14 and 0.48 and *p* value = 0.001, 0.02 and 3e-18 for the red, turquoise and yellow module respectively) and a negative correlation with the IGRA+ cohort (Pearson correlation coefficient = -0.22, -0.12 and -0.22 and *p* value = 1e-04, 0.04 and 1e-04 for the red, turquoise and yellow module respectively). The strongest correlation was between ATB at diagnosis and the yellow module (Pearson correlation coefficient = 0.48 and *p* value = 3e-18), and this module was also the only one showing a significant negative correlation with the IGRA- cohort (Pearson correlation coefficient = -0.26 and *p* value = 9e-06). The blue and green modules showed no significant correlation with any cohort.

We then specifically looked at how the expression of each module that significantly correlated with ATB at diagnosis varied across both CD14^+^ monocyte subsets. The brown module (negatively correlated with ATB at diagnosis) showed decreased expression between ATB diagnosis and IGRA+/- cohorts in both CD14^+^ monocyte populations (**Figure 2D**). Similarly, the yellow module (positively correlated with ATB at diagnosis) showed increased expression in ATB diagnosis compared to IGRA+/- cohorts in both CD14^+^ monocyte populations (**Figure 2E**). In contrast, the red and turquoise modules (also positively correlated with ATB at diagnosis) showed increased expression in ATB diagnosis compared to IGRA+/- cohorts in intermediate CD14^+^CD16^+^ monocytes only (**Figure 2F**). Looking at paired differences in ATB patients between diagnosis and mid-treatment samples, we observed a reduced expression of the yellow and turquoise modules at mid-treatment compared to diagnosis in both CD14^+^ monocyte subsets (**Figures 2E, F**), and an increased expression of the brown module in intermediate CD14^+^CD16^+^ monocytes only (**Figure 2D**).

Taken together, these data identified transcriptomic modules that could not only distinguish classical CD14^+^CD16^-^ from intermediate CD14^+^CD16^+^ monocytes, but also showed significant expression changes in ATB patients at diagnosis compared to the other cohorts. The intermediate CD14^+^CD16^+^ monocyte population showed the highest number of modules with differential expression in ATB at diagnosis compared to the other cohorts with three modules upregulated (yellow, turquoise and red) and one module downregulated (brown).

### In ATB at diagnosis, interferon signaling genes are upregulated in both classical CD14^+^CD16^-^ and intermediate CD14^+^CD16^+^ monocytes, and overlap with previously reported blood TB signatures

To further characterize the transcriptomic changes in CD14^+^ monocytes in ATB at diagnosis, we investigated the nature of the genes contained within the modules that showed significant expression changes in this cohort (i.e., brown, yellow, turquoise and red modules, as identified in **Figure 2**). We ran a biological pathway analysis on the top 50 weighed genes of each module (see **Table S4** for individual gene list), and selected the top 10 pathways for display (**Figure 2G**, full list in **Table S5**). The yellow module (which showed increased expression in both CD14^+^ monocyte subsets in ATB at diagnosis) was associated with the highest statistical significance for biological pathway enrichment, with a strong enrichment for both type I and type II interferon (IFN) signaling (**Figure 2G**). IFN signaling has been repeatedly associated with blood transcriptomic signatures of ATB, identified either for diagnostic or prognostic purposes (9, 11, 14, 16, 18). Specifically, 39 out of the top 50 genes in the yellow module (including 22 of the 26 genes associated with IFN signaling) were present in the seminal IFN-associated blood signature of ATB identified by Berry et al. (**Figure S2**) (18). Thus, our data demonstrate that both classical CD14^+^CD16^-^ and intermediate CD14^+^CD16^+^ monocytes contribute to the IFN-associated gene signature previously identified in the blood of ATB patients.

### Intermediate CD14^+^CD16^+^ monocytes in ATB patients at diagnosis displayed increased expression of inflammatory and MHC-II genes, and increased capacity to activate T cells

**Figure 3:**
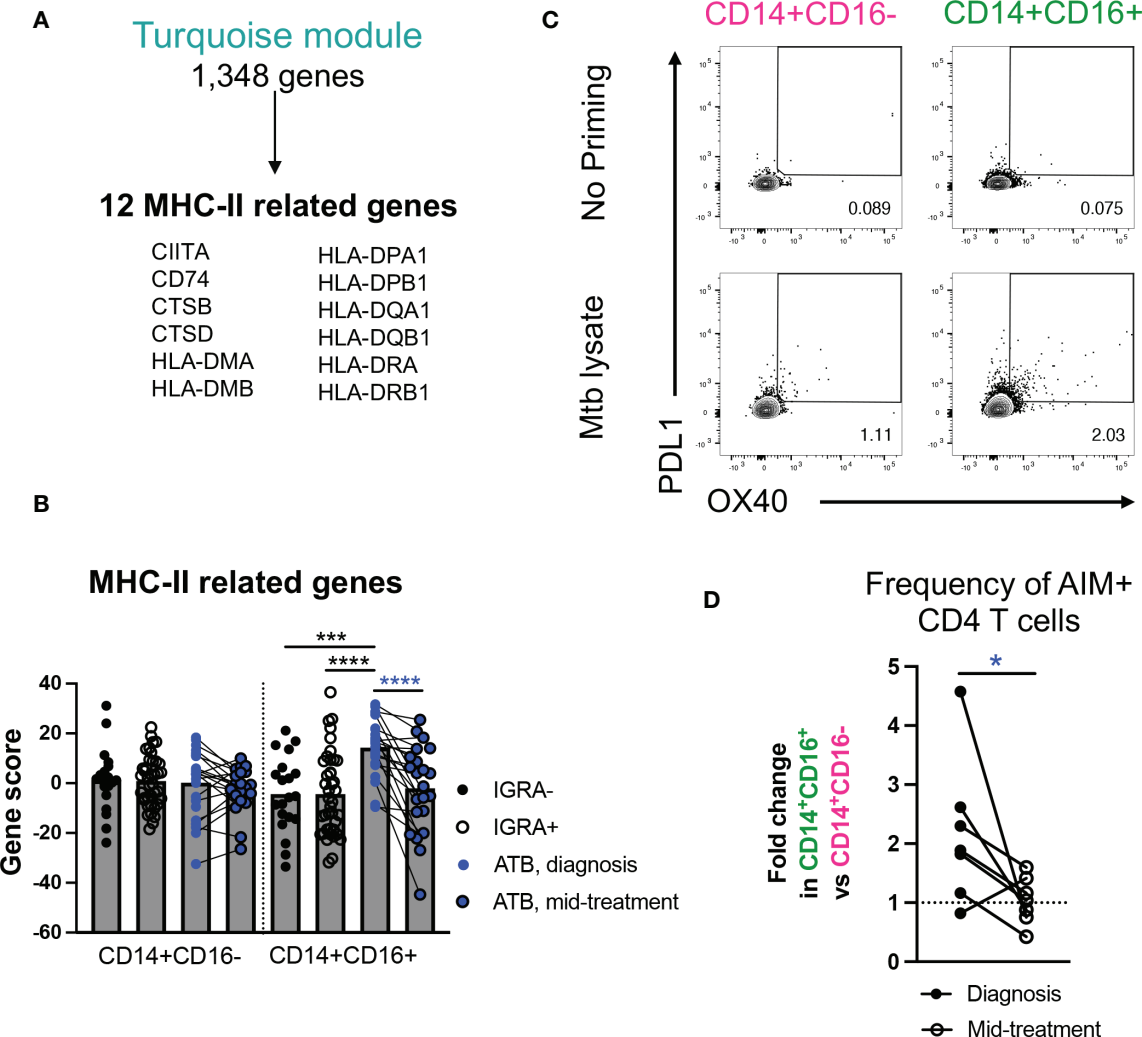
In ATB at diagnosis, intermediate CD14^+^CD16^+^ monocytes are associated with upregulation of MHC-II gene signatures and increased capacity to activate T cells. **(A)** MHC-II related genes present in the turquoise module. **(B)** Combined MHC-II gene expression in CD14^+^CD16^+^ and CD14^+^CD16^-^ cells in ATB diagnosis, ATB mid-treatment, IGRA+ and IGRA- cohorts, calculated using a standard z-score formula. Individual gene expression is shown in **Figure S3**. **(C)** Representative staining of OX40 and PDL1 expression in CD4 T cells after co-culture with autologous *Mtb*-primed or unprimed CD14^+^CD16^+^ and CD14^+^CD16^-^ cells sorted from one ATB subject at diagnosis. **(D)** Fold change in the frequency of OX40^+^PDL1^+^ cells in CD4 T cells after co-coculture with autologous *Mtb*-primed CD14^+^CD16^+^ cells vs. CD14^+^CD16^-^ cells sorted from ATB subjects at diagnosis, or mid-treatment. The frequency of OX40^+^PDL1^+^ CD4 T cells induced after co-culture with unprimed CD14^+^CD16^+^ or CD14^+^CD16^-^ cells was used for background subtraction. **(A, B)** Data was from cryopreserved PBMC of 25 ATB subjects at diagnosis (with 22 paired mid-treatment samples), 40 IGRA+ and 20 IGRA- individuals. **(C, D)** Data was from cryopreserved PBMC of 7 ATB subjects with paired samples collected at diagnosis and mid-treatment. *p < 0.05, ***p < 0.001, ****p < 0.0001, nonparametric unpaired Mann-Whitney U test (black stars) and nonparametric paired Wilcoxon test (blue stars).

Next, we focused on the gene expression changes specific to the intermediate CD14^+^CD16^+^ monocyte population, namely those associated with the turquoise and red modules. Both modules were enriched for pathways associated with metabolism (turquoise module: “glutathione metabolism”, “chondroitin sulfate/dermatan sulfate degradation”, “lipid and lipoprotein metabolism”, “pentose phosphate pathway”; red module: “PIP2 hydrolysis”, “glycerolipid metabolism”, “phosphoinositide signaling pathway”, “glycerophospholipid metabolism) (**Figure 2G**). In addition, the turquoise module was associated with i) inflammation (e.g., “leptin influence on immune response” (BST1, CD36, CSF3R, IL1RN, LYZ, NCF4, PTAFR) and “endogenous Toll-like receptor signaling” (CD14, S100A8, S100A9, VCAN)), and ii) antigen presentation (“cross-presentation of particulate endogenous antigens”) (**Figure 2G** and **Table S5**). It has previously been reported that, in steady state, intermediate CD14^+^CD16^+^ monocytes hold higher antigen presentation capability compared to classical CD14^+^CD16^-^ monocytes (30). To test whether this observation may also hold true in ATB, we looked more in depth for antigen presentation genes in our transcriptomic dataset. Within the 1,348 total genes present in the turquoise module (**Table S3**), we identified 12 genes related to MHC-II, including CIITA, CD74, and several HLA-DP, DQ, and DR genes (**Figure 3A**). Together, these 12 genes were significantly upregulated in intermediate CD14^+^CD16^+^ monocytes in ATB at diagnosis compared to IGRA+/- cohorts, but not in classical CD14^+^CD16^-^ monocytes (combined gene score expression **Figure 3B**, and individual gene expression **Figure S3**). All 12 MHC-II related genes were also significantly downregulated in intermediate CD14^+^CD16^+^ monocytes in ATB patients sampled at diagnosis compared to mid-treatment, whereas only CIITA, CTSB and CTSD showed significant reduction in expression upon treatment in classical CD14^+^CD16^-^ monocytes (**Figure S3**).

To elucidate whether the upregulation of inflammatory and MHC-II genes in intermediate CD14^+^CD16^+^ monocytes in ATB patients at diagnosis were associated with functional changes, we investigated the ability of this monocyte subset to activate T cells, in the presence or absence of *Mtb* antigens. Similar to our transcriptomic analysis, we sorted intermediate CD14^+^CD16^+^ monocytes from PBMC of ATB patients sampled at both diagnosis and mid-treatment (gating strategy **Figures 1A, C**), incubated them for five hours with *Mtb* lysate, and then added autologous sorted CD4 T cells from the mid-treatment sample (gating strategy **Figure S1C**). We elected to sort autologous T cells only from the mid-treatment sample to correct for potential changes in *Mtb*-specific T cell frequency and reactivity during treatment. After 24 hours of co-culture, we looked for the co-upregulation of Activation Induced Markers (AIM) OX40 and PD-L1 on the surface of T cells by flow cytometry. These markers were previously used to identify *Mtb*-reactive CD4 T cells after *in vitro* simulation (36). As a control, we also sorted classical CD14^+^CD16^-^ monocytes in the same ATB patients at both timepoints, which from our transcriptomic analysis did not show upregulation of MHC-II related genes as observed in intermediate CD14^+^CD16^+^ monocytes. For both CD14^+^ monocyte subsets, priming with *Mtb* lysate increased the frequency of AIM^+^ CD4 T cells in comparison to no antigen priming (**Figure 3C**). The frequency of AIM^+^ CD4 T cells induced with *Mtb*-priming was superior when using intermediate CD14^+^CD16^+^ monocytes compared to classical CD14^+^CD16^-^ monocytes (mean value of 2.9% vs 2.1% AIM+ CD4 T cells for intermediate monocytes vs classical monocytes, respectively), and this effect was significantly greater at diagnosis compared to mid-treatment (*p* value = 0.031, **Figure 3D**). Thus, intermediate CD14^+^CD16^+^ monocytes isolated from ATB patients at diagnosis have increased expression of genes associated with metabolism, inflammation and MHC-II, and increased capacity to activate T cells upon *Mtb* antigen exposure, and these characteristics dissipate following 2-3 months of TB therapy.

### Single-cell transcriptomics reveals the interferon, MHC-II, and inflammatory gene signatures originates from distinct subsets of intermediate CD14^+^CD16^+^ monocytes

**Figure 4:**
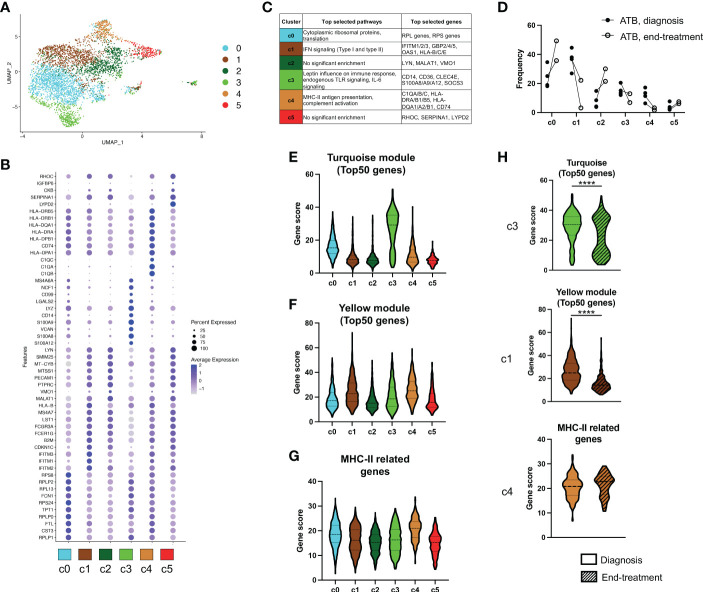
Multiple subsets of intermediate CD14^+^CD16^+^ monocytes are contributing to the transcriptomic signature of ATB at diagnosis. **(A)** UMAP analysis of single-cell RNA sequencing of CD14+CD16+ myeloid cells isolated from cryopreserved PBMC of ATB patients at diagnosis (n=4, visit 1) or end of treatment (n=2, visit 2, paired with visit 1). All cells were divided into six distinct clusters. **(B)** Dot plot showing the expression of the selected top 10 genes for each of the six clusters identified in A). **(C)** Selected top biological pathways enriched and top genes expressed for each cluster. Full list of genes and pathways for each cluster are available in **Table S6** and **S7**. **(D)** Individual cluster frequency for each ATB patient at diagnosis (Visit 1) and end of treatment (Visit 2). Expression of **(E)** The turquoise module, **(F)** The yellow module and **(G)** MHC-II related genes, in each cluster. **(H)** Expression of the signatures shown in E-G split by sample visit (diagnosis vs end-treatment). For each signature, expression was only shown for the cluster with highest expression as depicted in E-G (i.e., cluster 3 for the turquoise module, cluster 1 for the yellow module, and cluster 4 for MHC-II related genes). The turquoise module, yellow module and MHC-II related gene signatures were identified in our bulk RNA sequencing analysis as represented in **Figures 2** and **3**. Gene scores were calculated by summing all genes composing the signature, or the top 50 genes for the modules. ****p < 0.0001, nonparametric unpaired Mann-Whitney U test.

To further characterize the individual subsets responsible for our newly identified gene signatures of ATB at diagnosis in intermediate CD14^+^CD16^+^ monocytes, we analyzed this cell population using single-cell RNA sequencing (scRNAseq). Using the same gating strategy as for the bulk analysis, we sorted CD14^+^CD16^+^ cells from cryopreserved PBMC of four ATB patients sampled at diagnosis. For two of the patients, a PBMC sample collected at the end of treatment (i.e., standard six-month anti-TB therapy) was also processed simultaneously. Dimensionality reduction and clustering analysis identified 6 clusters within intermediate CD14^+^CD16^+^ monocytes (**Figure 4A**), and each sample contributed to all 6 clusters, confirming that there was no significant batch effect between samples (**Figure S5C**). Each cluster was associated with a distinct gene expression profile (**Figure 4B** and **Table S6**) and a significant enrichment for biological pathways was found for 4 out of 6 clusters (clusters 0, 1, 3 and 4; adjusted *p*-value < 0.05; **Figure 4C** and **Table S7**). Three of the six clusters contained genes or pathways that were previously identified in our bulk RNA analysis as differentially expressed in ATB at diagnosis compared to the other cohorts. Cluster 1 was associated with IFN signaling, cluster 3 was associated with the same inflammatory genes (i.e., CD14, CD36, S100A8, S100A9) and pathways (i.e., “leptin influence on immune response” and “endogenous TLR signaling”) identified in the turquoise module, and cluster 4 was enriched for MHC-II related genes (**Figure 4C**). Interestingly, these three clusters also showed reduced frequency upon treatment in both patients with paired sampling, whereas the other three clusters (clusters 0, 2 and 5) had an increased frequency upon treatment (**Figure 4D**).

To further look at the association between the bulk and single-cell transcriptomic datasets, we investigated the expression of each of the transcriptomic signatures derived from our bulk RNA sequencing modular analysis across the single-cell clusters. As expected from its top expressed genes and associated biological pathways, the ‘inflammation’ cluster 3 was associated with the highest cumulative expression for the turquoise module (**Figure 4E**). Similarly, cluster 1 and cluster 4 displayed the highest expression for the yellow module (which was associated with IFN signaling) and for MHC-II genes, respectively (**Figures 4F, G**), also matching the results from the biological pathway enrichment analysis. In both ATB patients with paired sampling, the turquoise module and the yellow module gene signatures showed a reduction in expression within their respective cluster between diagnosis and end-treatment, whereas the expression level of MHC-II related genes in cluster 4 remained unchanged upon treatment (**Figure 4H**).

Taken together, our results from the single-cell data analysis demonstrate that each signature of ATB at diagnosis derived from our bulk RNA sequencing analysis could be associated with a distinct subset of intermediate CD14^+^CD16^+^ monocytes. Clusters 1, 3 and 4 were responsible for the IFN, inflammatory, and MHC-II gene signatures, respectively.

## Discussion

Despite the importance of monocytes in blood immune responses to *Mtb* infection, no study has systematically looked at the global circulating monocyte compartment in the context of active disease (ATB), asymptomatic infection (IGRA+), and no infection (IGRA-). Here, we performed a comprehensive single-cell profiling of circulating monocytes from IGRA+/- healthy individuals and a longitudinal cohort of ATB patients at diagnosis/mid-treatment in order to characterize quantitative and qualitative changes associated with TB disease. This study represents the largest exploratory analysis of the monocyte compartment in TB published to date.

Several studies have shown increased M/L ratio in ATB patients before the start of anti-TB therapy and highlight the potential use of this parameter as a diagnostic tool (24, 37, 38). Here, we confirmed that M/L ratio was significantly increased in ATB patients at diagnosis, and subsequently decreased upon initiation of anti-TB therapy. These results also corroborate the usefulness of flow cytometry to measure M/L ratio within cryopreserved PBMC, in contrast to the traditional full blood count assay which can only be done on fresh blood, as previously suggested in other studies (39).

Incrementally deepening the cellular level of our analysis, we next identified that both classical CD14^+^CD16^-^ and intermediate CD14+CD16+ monocyte populations contributed to the dysregulated M/L ratio in ATB patients, with increased frequency at diagnosis and a reduction upon initiation of anti-TB therapy. This is in line with previous studies which have shown that ATB patients have an increased percentage of circulating CD14^+^CD16^+^ monocytes in ATB as compared to TST+/- healthy controls (40), and this effect is reduced following anti-TB treatment (41). In addition to changes in monocyte population frequencies, we also identified transcriptomic differences in both CD14^+^ monocyte populations in ATB patients at diagnosis, compared to IGRA+/- cohorts. Similarly to the M/L ratio and monocyte frequencies, these changes were transient, as they were no longer observed in the same ATB patients following two to three months of anti-TB therapy. In particular, we identified several transcriptomic modules with distinct expression levels across both CD14^+^ monocyte subsets that were dysregulated in ATB at diagnosis compared to the other cohorts. These modules were associated with distinct biologic pathways and functions.

We identified that the most prominent transcriptomic changes across both classical CD14^+^CD16^-^ and intermediate CD14^+^CD16^+^ monocytes were associated with IFN signaling (yellow module), and significantly overlapped with previously published whole blood gene signatures of ATB. Identified by Berry et al, the IFN gene signature in whole blood of ATB patients at diagnosis was shown to be mostly expressed by neutrophils, and to a lesser extent, CD14^+^ monocytes (18). Here we further show that, in ATB at diagnosis, both classical CD14^+^CD16^-^ and intermediate CD14^+^CD16^+^ monocytes express this signature. Since CD14^+^ monocytes are at increased frequency in the circulation in ATB patients at diagnosis, it is likely that they contribute to the IFN gene signature repeatedly observed in whole blood in this disease cohort. The presence of an IFN signature in monocytes from ATB patients has multiple biological implications. Both type I and type II IFN have been shown to play a key role during *Mtb* infection. Type I IFN can be produced by monocytes, macrophages, and DC through recognition of *Mtb* by a wide range of pattern recognition receptors (42). In ATB, there is abundant evidence that type I IFN are deleterious by promoting bacterial expansion and pathogenesis, but that they could also provide a protective effect to the host at low levels or in absence of IFNγ (42). In contrast, the IFNγ pathway is crucial for protection against *Mtb* (43). IFNγ is produced by *Mtb*-specific T cells, and may be also responsible for the activation of IFN signaling pathways in circulating immune cells expressing IFN receptors, such as monocytes, in an antigen-independent fashion. Finally, both type I and type II IFN drives emergency myelopoiesis and recruitment to the lung in an interplayed manner in ATB (44) and may thus be responsible for the overall higher M/L ratio we (and others) have observed in the blood of ATB patients.

In addition to IFN signaling, we identified in intermediate CD14^+^CD16^+^ monocytes several novel transcriptomic signatures that were specifically dysregulated in ATB patients. Specifically, intermediate CD14^+^CD16^+^ monocytes isolated from ATB patients showed increased expression of genes associated with metabolism, inflammation, and MHC-II. These changes were more prominent at diagnosis, and decreased upon initiation of anti-TB therapy. All three gene categories are known to be expressed by activated immune cells. Upon activation, immune cells undergo metabolic reprogramming, which is critical for their proliferation, differentiation and function (45). Similarly, the expression of inflammatory genes is a hallmark for immune cell activation, and antigen-presenting cells upregulate MHC-II genes upon activation (46). Finally, we observed that intermediate CD14^+^CD16^+^ monocytes isolated from ATB patients at diagnosis had increased capacity to activate T cells *in vitro* in the presence of *Mtb* antigens, in comparison to classical CD14^+^CD16^-^ monocytes. Taken together, our results demonstrate that in ATB at diagnosis, the circulating intermediate CD14^+^CD16^+^ monocyte population is not only increased in frequency, but is also in a heightened activation state.

To gain an unprecedented level of information on intermediate CD14^+^CD16^+^ monocytes in ATB at diagnosis, we further analyzed this cell population using single-cell transcriptomics. Surprisingly, our analysis identified that the IFN, the MHC-II and the inflammatory gene signatures identified in our bulk transcriptomic analysis were not co-expressed but rather carried by three distinct subsets of CD14^+^CD16^+^ cells. Thus, we identified novel subsets of CD14^+^CD16^+^ monocytes with distinct transcriptomic signatures and associated biological pathways that may hold important immune functions in ATB. The protective role of each subset and their relationship to each other, how their frequency and phenotype may vary upon treatment, vaccination and in other TB cohorts such as resistors and progressors, are yet to be defined. Indeed, it was previously found that ATB diabetic patients have a higher level of monocyte activation markers in their plasma compared to ATB only, and that circulating monocytes have reduced HLA-DR expression in diabetic patients. But together, our data illustrate the heterogeneity of intermediate CD14^+^CD16^+^ monocytes in human blood and suggest that each subset may hold distinct immune functions in ATB.

As reviewed in our introduction, the myeloid population present in PBMC with a CD14^+^CD16^+^ phenotype is expected to be intermediate monocytes. A recent single-cell transcriptomic study highlighted the limitation of this phenotypic definition by demonstrating high heterogeneity of human blood myeloid cells, with subsets of DC and monocytes presenting phenotypic and transcriptomic overlaps, such as the expression of CD14 (47). DC are also generally regarded as professional antigen presenting cells, with superior antigen presentation capability and constitutive high expression of MHC-II genes in comparison to monocytes (48). Here, we observed that in ATB patients at diagnosis, intermediate CD14^+^CD16^+^ monocytes upregulated several MHC-II related genes and hold increased capability to activate T cells upon antigen exposure. Additionally, the inflammatory gene signature we identified as upregulated in a subset of intermediate CD14^+^CD16^+^ monocytes in ATB patients at diagnosis significantly overlapped with the gene signature of a pro-inflammatory subset of DC3, namely CD14^+^CD163^+^ DC3, as recently reported in a comprehensive single-cell high dimensional analysis of myeloid cell subsets in human blood (47) (*p* value of overlap = 4.1e-11, **Figure S4A**). Specifically, CD163 gene expression was strongly upregulated in ATB patients at diagnosis compared to both IGRA+ and IGRA- cohorts in CD14^+^CD16^+^ but not CD14^+^CD16^-^ cells (**Figure S4B**). Furthermore, the gene signature of CD14^+^CD163^+^ DC3 defined by Dutertre et al. (47) could separate ATB diagnosis from IGRA+/- cohorts in intermediate CD14^+^CD16^+^ monocytes, but not in classical CD14^+^CD16^-^ monocytes (**Figure S4C**), and showed the highest cumulative expression in the inflammatory cluster 3 from the single-cell analysis of CD14^+^CD16^+^ cells in ATB (**Figure S4D**). Based on these observations, a logical question arising would be whether subsets of DC, in particular pro-inflammatory DC3, could be present in the intermediate CD14^+^CD16^+^ monocyte population in ATB patients at diagnosis, and contribute to the MHC-II and inflammatory gene signatures identified herein. Dutertre et al. identified CD88 (C5AR1) and FCER1A as constitutive and specific markers of monocytes and DC, respectively (47). In our scRNAseq dataset, we found little to no expression of FCER1A and uniform expression of C5AR1 across all 6 clusters of CD14^+^CD16^+^ myeloid cells (**Figures S4E, F**). Thus, despite transcriptomic and functional overlaps with subsets of DC, the inflammatory and MHC-II subsets within CD14^+^CD16^+^ myeloid cells isolated from ATB patients at diagnosis are *bona fide* intermediate monocytes and not DC.

In conclusion, we demonstrated that quantitative and qualitative changes are occurring in circulating monocytes during ATB. We showed that the increase in M/L ratio in ATB at diagnosis stemmed from classical CD14^+^CD16^-^ and intermediate CD14^+^CD16^+^ monocytes, and that the most prominent transcriptomic changes in these two monocyte subsets were upregulation of IFN-associated genes that significantly overlapped with previously characterized blood signatures of ATB. Additional transcriptomic and functional changes were present in intermediate CD14^+^CD16^+^ monocytes in ATB at diagnosis, such as the expression of inflammatory genes, MHC-II genes, and increased capacity to activate T cells, overall reflecting a more prominent activation in this monocyte population. Single-cell transcriptomics revealed that distinct subsets of intermediate CD14^+^CD16^+^ monocytes were responsible for each of these signatures. Together, our study demonstrates the heterogeneity of circulating CD14^+^ monocytes and their important contribution to blood immune signatures in ATB.

## Material and methods

### Participants and samples

Cohorts’ description and demographics is available in **Table S1** *. Mtb* sensitization status was confirmed in participants by a positive IFNγ–release assay (QuantiFERON-TB Gold In-Tube; Cellestis or T-SPOT.TB; Oxford Immunotec) and the absence of symptoms consistent with TB, or other clinical/radiographic signs of ATB (healthy IGRA+ cohort). ATB was defined as 1) presence of clinical symptoms and/or radiological/histological evidence of pulmonary TB, and 2) microbiologically confirmed by *Mtb*-specific molecular testing on sputum. IGRA- uninfected controls had no past medical history of TB, nor exposure to *Mtb* or evidence of *Mtb* sensitization as confirmed by a negative IFNγ–release assay. All participants were confirmed negative for human immunodeficiency virus (HIV) infection. For ATB subjects, blood samples were obtained at diagnosis and at 2-3 months upon initiation of anti-TB therapy. Anti-TB therapy was a standard regimen for drug susceptible *Mtb* consisting of an intensive phase of 2 months of isoniazid (INH), rifampin (RIF), pyrazinamide (PZA), and ethambutol (EMB) followed by a continuation phase of 4 months of INH and RIF (49). Peripheral blood mononuclear cells (PBMC) were obtained by density gradient centrifugation (Ficoll-Hypaque, GE Healthcare) from leukapheresis or whole-blood samples, according to the manufacturer’s instructions. Cells were resuspended at 50–100 million cells per milliliter in FBS (Gemini Bio-Products) containing 10% DMSO (Sigma) and cryopreserved in liquid nitrogen.

### PBMC thawing

Cryopreserved PBMC were quickly thawed by incubating each cryovial at 37°C for 2 min, and cells transferred into 9 ml of cold medium (RPMI 1640 with L-Glutamine and 25 mM Hepes (Omega Scientific), supplemented with 5% human AB serum (GemCell), 1% Penicillin Streptomycin (Gibco), 1% Glutamax (Gibco)), and 20 U/mL Benzonase Nuclease (Millipore). Cells were centrifuged and resuspended in medium to determine cell concentration and viability using Trypan blue and a hematocytometer. Cells were then kept at 4°C until use for flow cytometry or cell sorting.

### Flow cytometry

Flow cytometry experiments were performed as previously described (36, 50). For surface staining, up to 0.5x10^6^ cells were incubated with 10% FBS in 1X PBS for 10 minutes. Cells were then stained with 100 μl of PBS containing 0.1 μl fixable viability dye eFluor506 (eBioscience, corresponding to 1:1000 dilution of the stock, as per the manufacturer’s recommendation), 2 μl of FcR blocking reagent (Biolegend, corresponding to 1:50 dilution of the stock; we validated internally that this dilution is performing equally to the manufacturer’s recommended dilution of 1:20), and various combinations of the antibodies listed in **Table S2** for 20 min at room temperature. For single-cell RNA sequencing, TotalSeq™-C oligonucleotide-conjugated antibodies (Biolegend) were also added at this step at 0.01mg/mL final concentration. After two washes in PBS, cells were resuspended into 100 μl of MACS buffer (PBS containing 2mM EDTA (pH 8.0) and 0.5% BSA) and stored at 4°C protected from light for up to 4 hours until flow cytometry acquisition.

### Cell sorting

After PBMC thawing, 10x10^6^ cells were stained with fixable viability dye eFluor 506 (eBioscience) and with anti-human CD56, CD19, CD3, CD14 and CD16 (**Table S2** for antibody details), as described in the flow cytometry section above. Cell sorting was performed on a BD FACSAria III cell sorter (Becton Dickinson). Lineage negative CD14^+^CD16^-^ and CD14^+^CD16^+^ monocytes were identified as described in **Figures 1A, C**. For bulk RNA sequencing, a total of 100,000 cells for each population was sorted into TRIzol LS reagent (Invitrogen) and used for bulk RNA sequencing. For single-cell RNA sequencing, 15,000 cells of each cell population were sorted into low-retention 1.5-ml collection tubes (Thermo Fisher Scientific), containing 0.5 ml of a 1:1 solution of phosphate-buffered saline (PBS):FBS supplemented with ribonuclease inhibitor (1:100; Takara Bio). For the T cell antigen presentation assay, up to 20,000 cells for each CD14^+^ monocyte subset (gating strategy **Figure 1A, C**) and up to 4 x 10^6^ CD4 T cells (defined as CD3^+^CD4^+^CD8^-^ in the live singlet gate population, see gating strategy **Figure S1C**) were sorted in MACS buffer (PBS containing 2mM EDTA (pH 8.0) and 0.5% BSA) and kept on ice until *in vitro* culture.

### T cell antigen presentation assay

Immediately following sorting, CD4 T cells were plated at 1 x 10^6^ cells per well in HR5 media (RPMI 1640 containing 5% human serum, 1% GlutaMAX 100x (Gibco), and 1% Pen-Strep) and each well was supplemented with 0.02 U/μL of recombinant human IL-2 (Prospec). In parallel, sorted CD14^+^CD16^-^ and CD14^+^CD16^+^ myeloid cells were resuspended in HR5 media and plated separately at 10,000 cells per well. Both CD4 T cells and myeloid cells cultures were incubated at 37°C. At the 19-hour timepoint, myeloid cells were primed with 10 μg/mL of whole cell *Mtb* lysate (strain H37Rv, BEI Resources) or left unprimed. At the 24-hour timepoint, CD4 T cells were resuspended in fresh HR5 media, and added to myeloid cells in a 1:25 (myeloid to T cell) ratio and co-cultures were further incubated for 24 hours at 37°C. At the 48-hour timepoint, cells were stained for flow cytometry, as described above, for T cell lineage markers CD3 and CD4, and T cell activation markers OX40 and PD-L1 (antibody list in **Table S2**).

### Bulk RNA sequencing

RNA sequencing was performed as described previously (36). In brief, total RNA was purified using an miRNeasy Micro Kit (QIAGEN) and quantified by quantitative PCR, as described previously (51). Purified total RNA (1–5 ng) was amplified following the Smart-Seq2 protocol (16 cycles of cDNA amplification) (52). cDNA was purified using AMPure XP beads (Beckman Coulter). From this step, 1 ng of cDNA was used to prepare a standard Nextera XT sequencing library (Nextera XT DNA sample preparation kit and index kit, Illumina). Whole-transcriptome amplification and sequencing library preparations were performed in a 96-well format to reduce assay-to-assay variability. Quality-control steps were included to determine total RNA quality and quantity, the optimal number of PCR preamplification cycles, and fragment size selection. Samples that failed quality control were eliminated from further downstream steps. Barcoded Illumina sequencing libraries (Nextera; Illumina) were generated using the automated platform (Biomek FXp). Libraries were sequenced on a HiSeq 2500 Illumina platform to obtain 50-bp single-end reads (TruSeq Rapid kit; Illumina). Mapping was performed as previously described (36). Briefly, the single-end reads that passed Illumina filters were filtered for reads aligning to tRNA, rRNA, adapter sequences, and spike-in controls. The reads were then aligned to UCSC hg19 reference genome using TopHat (v 1.4.1) (53). DUST scores were calculated with PRINSEQ Lite (v 0.20.3) (54), and low-complexity reads (DUST>4) were removed from the BAM files. The alignment results were parsed *via* SAMtools (55) to generate SAM files. Read counts for each genomic feature were obtained with the htseq-count program (v 0.6.0) (56) using the “union” option. After removing absent features (zero counts in all samples), the raw counts were imported to R/Bioconductor package DESeq2 (57) to identify differentially expressed genes among samples.

### Bulk RNA sequencing analysis

Raw counts were filtered to remove outlier samples, as well as any genes that had an average TPM of less than 1. Normalization was then performed using DESeq2 (57) and data was transformed by variance stabilizing transformation. The SVA ComBat package was used to correct for batch effects. Differential expression analysis was performed using DESeq2 (57). For the modular analysis, normalized counts were sorted by COV and the top 3,000 genes with highest variation were selected, and gene modules identified with the R package WGCNA (35). A power of 17 was used when creating the adjacency matrix and the module distance threshold was 0.15. To determine the top 50 genes for each module, hub genes with high intramodular connectivity, i.e., genes that tend to have high correlation with other genes within the module, were identified with a minimum correlation of 0.75, and the top 50 of these hub genes from each module were retained. For each module, a module eigengene value was also calculated for each sample, summarizing the expression of all genes within that module for a sample into a single point representing the first principal component. Statistical enrichment for biological pathways was performed by interrogating the BioPlanet database (2019 version) using the online server Enrichr. Principal Component Analysis (PCA) and heatmaps were performed using vst normalized expression values using R and the software Qlucore. MHC-II related gene score was calculated for each sample using a standard z-score formula, by summing the normalized expression values for all MHC-II related genes identified in the turquoise module, and correcting for the average and standard deviation of the sums across all samples, as well as the number of samples.

### Single-cell RNA sequencing

Single-cell RNA sequencing was performed using the droplet based 10x genomics platform according to the manufacturer’s instructions. Lineage negative CD14^+^CD16^-^ and CD14^+^CD16^+^ monocytes (15,000 cells per population) were sorted from 6 different PBMC samples (4 ATB patients at diagnosis, including two with a paired sample collected at end of treatment) and pooled together. Each PBMC sample was stained with a distinct hashtag oligonucleotide antibody as described in the flow cytometry section, in order to determinate the sample origin for each cell after sequencing. Following cell sorting, cells were washed with ice-cold PBS, centrifuged for 10 min (600g at 4°C), gently resuspended in ice-cold PBS supplemented with 0.04% ultrapure bovine serum albumin (Sigma-Aldrich). The library preparation was performed using a 10x Genomics 5′ Tag v2 chemistry kit with dual indexes and feature barcoding technology for cell surface proteins. The amplification of complementary DNA was carried out with 13 cycles of amplification; the 5′ Tag gene expression libraries and the corresponding hashtag libraries were generated separately with 13 and 8 cycles of amplification, respectively. The libraries were sequenced using the Illumina NovaSeq 6000 sequencing platform with the following read lengths: read 1, 101 cycles; read 2, 101 cycles; i7 index, 10 cycles; i5 index, 10 cycles.

### Single-cell RNA sequencing analysis

The reads from the scRNAseq library were demultiplexed, aligned, and collapsed into Unique Molecular Identifier (UMI) counts using the software cell Ranger (v5.0.0) from 10x Genomics and the human genome reference GRCh38 (GENCODE v32/Ensembl 98). Mapped read counts were then analyzed using the Seurat package (v4.0.2) in R (58). Sample barcode assignment to each cell was performed using the HTO demultiplexing function (HTOdemux), and events classified as “negative” or “doublet” were excluded. To further eliminate intraindividual doublets and cells with low quality RNA, only HTO classified “singlet” cells with a percentage of mitochondrial genes lower than 6%, a total number of genes comprised between 500 and 3,500, and a total number of reads lower than 10,000 were retained. Normalization was performed using the SCTransform function, and correcting for batch effect across samples. Dimensionality reduction and clustering analysis was performed with the following command lines and parameters (runPCA: npcs = 50; FindNeighbors: dims = 1:30, k.param = 200; FindClusters: resolution = 0.6; RunUMAP: dims = 1:30). Our initial analysis identified a significant proportion of lymphoid-like cells (based on positive expression of CD2, CD3 and lack of C5AR1 expression), fairly distant from the remaining group of cells (cluster 2 in **Figure S5A**). Since our analysis was exclusively focused on myeloid cells, we excluded this cell cluster in our downstream analysis. Myeloid-like cells were then separated into CD16+ and CD16- subsets based on FCGR3A expression (**Figure S5B**), yielding a similar number of cells in both groups (as expected, since a similar number of CD14^+^CD16^-^ and CD14^+^CD16^+^ cells were initially sorted and pooled together for sequencing). Dimensionality reduction and clustering analysis of CD14^+^CD16^+^ cells was performed with the same command lines and parameters as described for our initial analysis of all cells, with the exception of the RunClusters resolution adjusted to 1. Top genes for each cluster were extracted using the FindAllMarkers function with parameters min.pct = 0.25, logfc.threshold = 0.25, return.thresh = 0.05, test.use = ‘MAST’, selecting only the positive genes. Graphic visualization of the results (UMAP plots, violin plots and dot expression plots) were all performed with the Seurat package (v4.0.2) in R (58).

### Statistics

Statistical analyses were performed using GraphPad Prism Software, version 9. Paired datasets were compared using the nonparametric Wilcoxon test, while unpaired datasets were compared using the nonparametric Mann-Whitney U test. P values less than 0.05 were considered significant and 2-tailed analyses were performed. Statistical significance of overlap between the top 50 gene list in the turquoise module and the previously reported CD14^+^CD163^+^ DC3 gene signature was calculated using the hypergeometric distribution test and considering all 15,643 genes that were detected across CD14^+^CD16^-^ and CD14^+^CD16^+^ cell populations as the total number of genes.

### Study approval

Human study participants were enrolled at the University of California, San Diego Anti-Viral Research Center clinic (United States), the National Hospital for Respiratory Diseases, Welisara (Sri Lanka), or The South African Tuberculosis Vaccine Initiative, University of Cape Town, Western Cape Province (South Africa). Ethical approval to carry out this work was maintained through the La Jolla Institute for Immunology Institutional Review Board (IRB) or the Human research Ethics Committee of the University of Cape Town. The University of Colombo Ethics Review Committee served as the National Institute of Health registered IRB for the Kotelawala Defence University, to collect samples from the National Hospital for Respiratory Diseases, Welisara (Sri Lanka). All clinical investigations were conducted according to the principles expressed in the Declaration of Helsinki, and all participants provided written informed consent prior to participation in the study. All samples were obtained for specific use in this study.

## Data availability statement

The sequencing data presented in this study were submitted to the Gene Expression Omnibus and are available under accession number GSE185372 and GSE214237 (https://www.ncbi.nlm.nih.gov/geo).

## Ethics statement

Ethical approval to carry out this work was maintained through the La Jolla Institute for Immunology Institutional Review Board (IRB) or the Human research Ethics Committee of the University of Cape Town. The University of Colombo Ethics Review Committee served as the National Institute of Health registered IRB for the Kotelawala Defence University, to collect samples from the National Hospital for Respiratory Diseases, Welisara (Sri Lanka). The patients/participants provided their written informed consent to participate in this study.

## Author contributions

BP and JB conceived and designed the study, with critical input from FS and CH. HH, NK, RTi, GS, PV and JB conducted the experiments. HH, NK, AS, PD and JB performed data analysis. AS, BG, JP, TS, CO, MF, AL and RTa provided clinical samples. BP and JB verified the underlying data and led the data interpretation with input from all authors. HH, BP and JB wrote the manuscript, and all authors edited the manuscript. All authors contributed to the article and approved the submitted version.
