# Supplementary material for: Single-cell profiling reveals distinct subsets of CD14+ monocytes drive blood immune signatures of active tuberculosis
Source: Front Immunol. 2023 Jan 11;13:1087010. doi: 10.3389/fimmu.2022.1087010 (PMC9874319; doi:10.3389/fimmu.2022.1087010)
Supplement: Supplementary file 1 [file DataSheet_1.pdf]

Supplementary material for

Single-cell profiling reveals distinct subsets of CD14+ monocytes drive the blood immune signature of active tuberculosis.

Hannah Hillman[1], Nabeela Khan[1], Akul Singhania[1], Paige Dubelko[1], Ferran Soldevila[1], Rashmi Tippalagama[1], Aruna D DeSilva[1,2], Bandu Gunasena[3], Judy Perera[2], Thomas J Scriba[4], Cynthia Ontong[4], Michelle Fisher[4], Angelique Luabeya[4], Randy Taplitz[5], Gregory Seumois[1], Pandurangan Vijayanand[1,6], Catherine C Hedrick[7], Bjoern Peters[1,6,*], Julie G Burel[1,*]

List of supplementary tables

Table S1: Cohorts' demographics

Table S2: Fluorochrome-conjugated antibodies used in the study

Table S3: Module classification for all 3,000 variable genes included in the modular analysis

Table S4: Top50 genes for each module

Table S5: Biological pathways significantly enriched for modules with dysregulated expression in ATB at diagnosis

Table S6: Genes significantly upregulated in each cluster of intermediate $CD14^{+}CD16^{+}$ monocytes.

Table S7: Biological pathways significantly enriched in each cluster of intermediate $CD14^{+}CD16^{+}$ monocytes.

Supplementary Figures

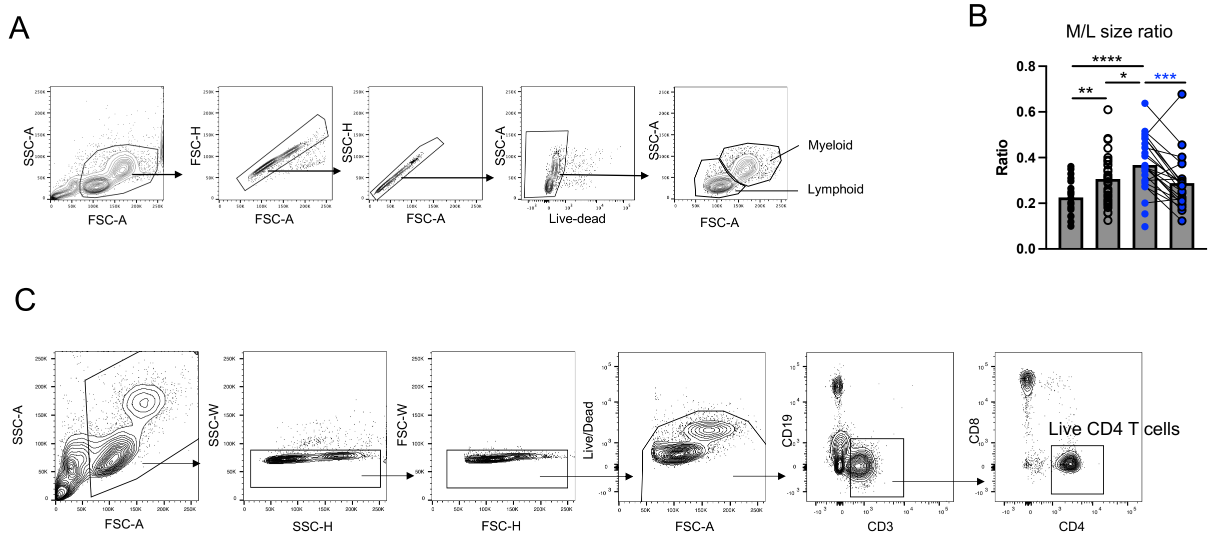

Figure S1: Gating strategies to identify myeloid and lymphoid cells from cryopreserved PBMC by flow cytometry. A) Identification of myeloid and lymphoid cell populations based on size and granularity (FSC and SSC parameters). **B**) M/L size ratio across ATB diagnosis, ATB mid-treatment, IGRA+ and IGRA- cohorts (calculated based on the myeloid and lymphoid populations identified in A). Data was from cryopreserved PBMC of 25 ATB subjects at diagnosis (with 22 paired mid-treatment samples), 40 IGRA+ and 20 IGRA- individuals. * $p < 0.05$, ** $p < 0.01$, *** $p < 0.001$, **** $p < 0.0001$, nonparametric unpaired Mann-Whitney U test (black stars) and nonparametric paired Wilcoxon test (blue stars). **C**) Gating strategy to identify CD4 T cells for the *in vitro* stimulation assay.

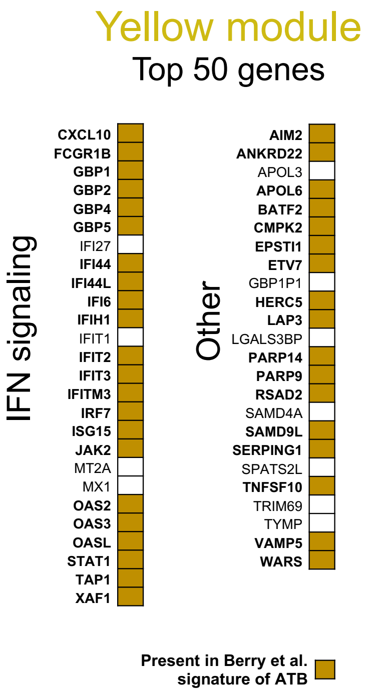

Figure S2: Overlap between the top 50 genes in the yellow module (associated with IFN signaling) and the seminal IFN-associated gene signature of ATB previously identified in blood by Berry et al (18).

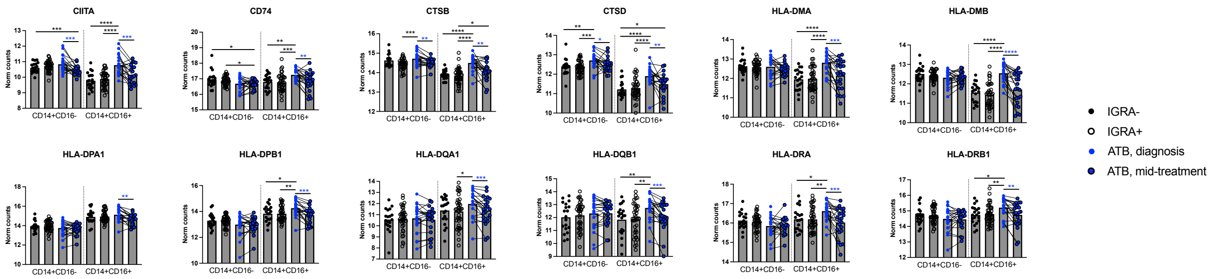

Figure S3: Individual gene expression of MHC-II related genes in sorted CD14^{+}CD16^{-} and CD14^{+}CD16^{+} cells in ATB, IGRA+ and IGRA- cohorts. Data was from cryopreserved PBMC of 25 ATB subjects at diagnosis (with 22 paired mid-treatment samples), 40 IGRA+ and 20 IGRA- individuals. * $p < 0.05$, ** $p < 0.01$, *** $p < 0.001$, **** $p < 0.0001$, nonparametric unpaired Mann-Whitney U test (black stars) and nonparametric paired Wilcoxon test (blue stars).

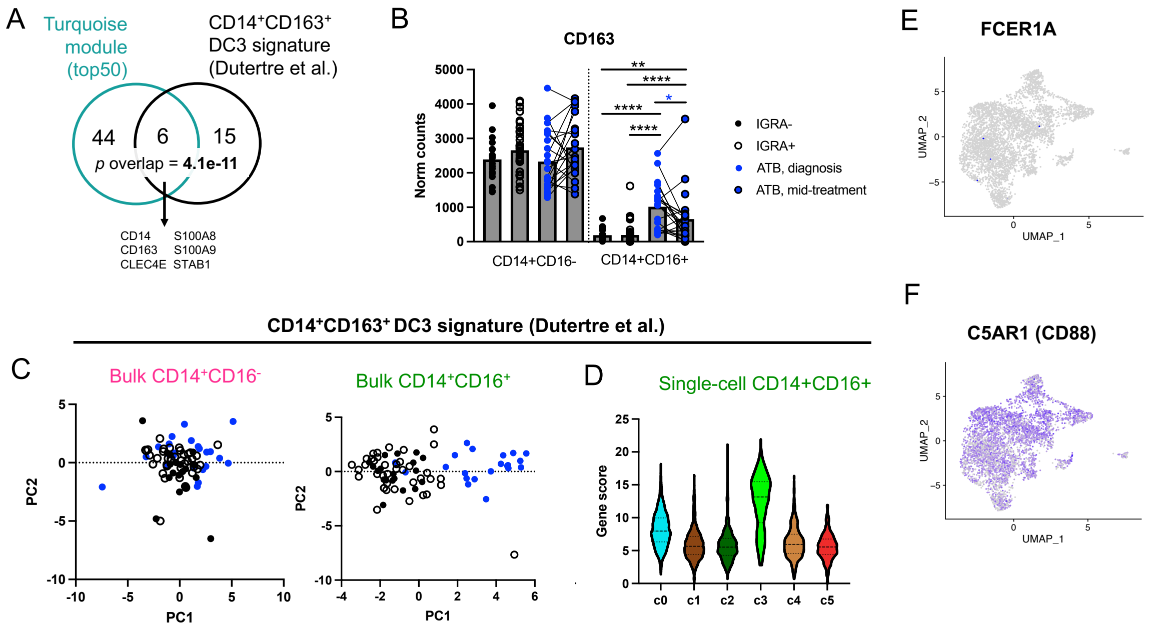

Figure S4: CD14⁺CD16⁺ myeloid cells in ATB at diagnosis display transcriptomic overlaps with a subset of pro-inflammatory dendritic cells but are *bona fide* monocytes

A) Overlap between the top 50 genes in the turquoise module and the 20-gene signature of CD14$^+$CD163$^+$ DC3 identified by single-cell RNA sequencing in (46). **B**) Principal component analysis of the 20-gene signature of CD14$^+$CD163$^+$ DC3 identified by Dutertre et al (46) applied to the transcriptome of CD14$^+$CD16$^+$ and CD14$^+$CD16$^-$ cells in ATB diagnosis, IGRA+ and IGRA- cohorts. **C**) Expression of the pro-inflammatory DC3 signature identified by Dutertre et al (46) amongst all six clusters identified in the single-cell RNA sequencing analysis of CD14$^+$CD16$^+$ cells, as depicted in Figure 4. Gene scores were calculated by summing all genes composing the signature. Feature UMAP plots showing the expression of **D**) the DC lineage marker FCER1A and **E**) the monocyte lineage marker C5AR1 (CD88) in all CD14$^+$CD16$^+$ cells. (A-B) Data was from cryopreserved PBMC of 25 ATB subjects at diagnosis (with 22 paired mid-treatment samples), 40 IGRA+ and 20 IGRA- individuals. (C-E) Data was from single-cell RNA sequencing of

$CD14^+CD16^+$ myeloid cells isolated from cryopreserved PBMC of ATB patients at diagnosis (n=4, visit 1) or end of treatment (n=2, visit 2, paired with visit 1).

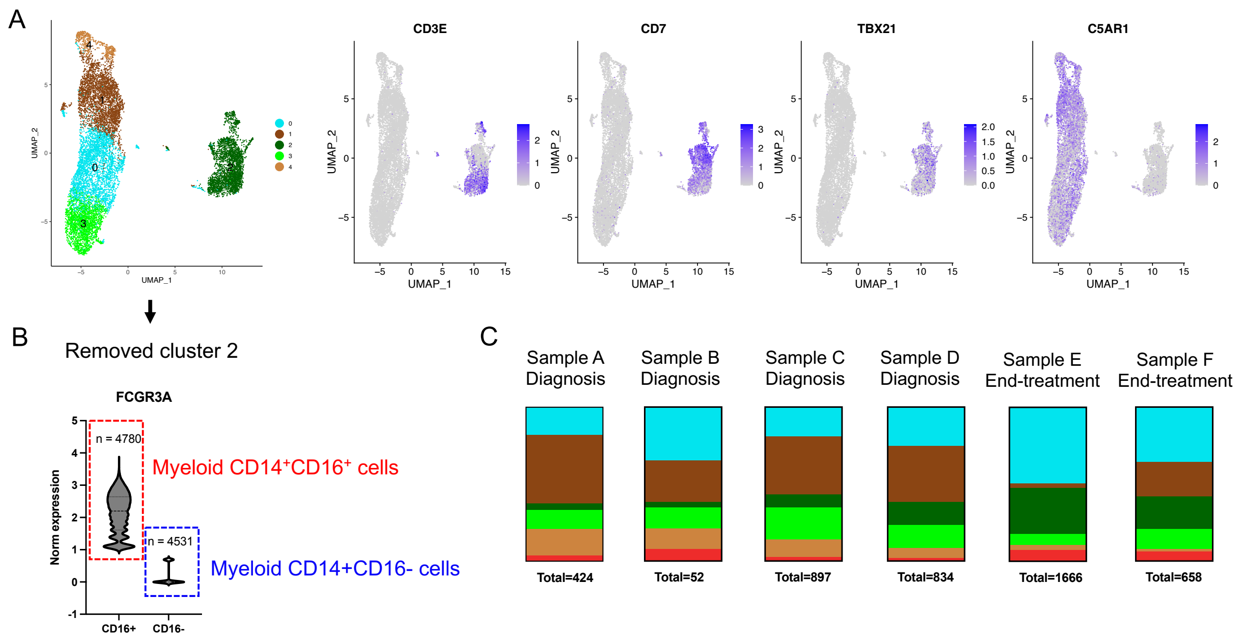

Figure S5: Single-cell RNA sequencing analysis of sorted lineage negative CD14+CD16- and CD14+CD16+ cells from PBMC of ATB patients. A) UMAP analysis of sorted CD14+CD16- and CD14+CD16+ myeloid cells isolated from cryopreserved PBMC of ATB patients at diagnosis (n=4, visit 1) or end of treatment (n=2, visit 2, paired with visit 1). All cells were divided into five distinct clusters. Cluster 2 was the most distant from the other clusters and showed positive expression of lymphoid markers such as CD3E, CD7 and TBX21 but no expression of the general myeloid marker C5AR1. **B)** Division of cells into CD16+ and CD16- subsets based on FCGR3A expression, after removing the lymphoid-like cells (cluster 2 in A). **C**) Cluster composition for each sample when analyzing CD16+ myeloid cells only (as shown in Figure 4A).
